# Older adults’ experiences of participating in longitudinal ageing research: a qualitative study from the Swedish National study on Aging and Care in Kungsholmen

**DOI:** 10.1136/bmjopen-2026-123537

**Published:** 2026-11-10

**Authors:** Nathalie Frisendahl, Ann Liljas, Mariam Kirvalidze, Janne Agerholm, Amaia Calderón-Larrañaga, Anna-Karin Welmer

**Affiliations:** 1 Department of Neurobiology, Care Sciences and Society, Karolinska Institutet, Stockholm, Sweden; 2 Department of Global Public Health, Karolinska Institutet, Stockholm, Sweden; 3 Aging Research Center, Department of Neurobiology, Care Sciences and Society, Karolinska Institutet and Stockholm University, Stockholm, Sweden; 4 Stockholm Gerontology Research Center, Stockholm, Sweden

**Keywords:** QUALITATIVE RESEARCH, PUBLIC HEALTH, Longitudinal studies

## Abstract

**Abstract:**

**Objectives:**

To explore older adults’ experiences of long-term participation in the Swedish National study on Aging and Care in Kungsholmen (SNAC-K), a population-based longitudinal study.

**Design:**

Exploratory qualitative study using focus group interviews.

**Setting:**

Population-based cohort study conducted in Kungsholmen, Stockholm, Sweden.

**Participants:**

Thirty-four older adults participating in the SNAC-K cohort were purposively sampled to ensure variation in age, gender and educational level. Data were collected between December 2023 and January 2024 and analysed using inductive qualitative content analysis.

**Results:**

Two main categories were identified: *blurred boundaries between research and health care* and *participation in research as meaningful yet demanding*. Participants valued the comprehensive examinations and repeated follow-ups, which enhanced their awareness and understanding of health and ageing. However, many also expressed expectations regarding feedback, reassurance and clinical follow-up, suggesting that the perceived value of participation may partly reflect unmet needs within routine healthcare provision. Participation in SNAC-K was generally experienced as meaningful and socially valuable, providing a sense of contributing to research and society, although cognitive testing and lengthy assessments were sometimes perceived as burdensome.

**Conclusions:**

Long-term participation in ageing research is experienced as both valuable and complex. Clear communication about the aims, scope and feedback procedures of observational studies may help align participants’ expectations and reduce misunderstandings regarding the roles of research and health care. Incorporating participants’ perspectives may further strengthen the ethical conduct and societal relevance of longitudinal ageing research.

**STRENGTHS AND LIMITATIONS OF THIS STUDY**

- Inclusion of participants with varied ages, gender and educational backgrounds enhanced the diversity of perspectives.
- Focus group discussions may have limited the expression of sensitive or critical experiences.
- Findings may have limited transferability to settings beyond similar urban and socioeconomically advantaged settings.

## Background

 Healthy ageing, as defined by the WHO,^1^ refers to the process of developing and maintaining the functional ability that enables well-being in older age. This ability results from the interaction between individuals’ intrinsic capacities and the physical and social environments in which they live. Given the complexity of factors influencing health and independence in later life, population-based longitudinal studies have become essential to advance understanding of ageing as a dynamic and multifactorial process. Population-based cohorts such as the Swedish National study on Aging and Care in Kungsholmen (SNAC-K) offer unique opportunities to capture this complexity by integrating health, social and care utilisation data over time.^2^ While such studies have generated extensive quantitative knowledge on ageing trajectories, they have traditionally focused on outcomes and determinants, with less attention to the experiences of the individuals who participate in them.

Participants play a central role in generating knowledge within longitudinal ageing research, yet older adults’ own perspectives on research participation remain relatively understudied.^3 4^ In recent years, there has been growing recognition of the importance of involving patients and members of the public more actively throughout the research process, both to improve the scientific and societal relevance of research and to strengthen its ethical conduct.^5–7^ Understanding participants’ perspectives may also contribute to improved study design, increased participation and reduced participant burden. However, participant perspectives within large-scale population-based longitudinal ageing studies have received limited attention.^3 4 8^ Existing studies suggest that older adults often view their involvement as meaningful and worthwhile, motivated by opportunities to monitor their health and contribute to future generations,^3 4^ but there remains a need for more in-depth qualitative research exploring how such experiences are understood and negotiated over time, particularly within ageing cohorts.

The aim of this qualitative study was therefore to explore older adults’ experiences of their long-term participation in the ongoing SNAC-K study, including the meaning they attribute to participation, their motivations for continued involvement, and their perceptions of contributing to research and society.

## Methods

### Study design

This is an exploratory qualitative study based on focus group interviews conducted in Stockholm, Sweden. The study followed the Standards for Reporting Qualitative Research (SRQR) checklist (online supplemental file 1).

A qualitative study design was applied, with data collected through focus group interviews.^9–11^ Group discussions can encourage interaction, reflection and engagement, thereby providing a rich and multifaceted understanding of participants’ perceptions of being involved in SNAC-K. This approach was also considered appropriate since all participants shared a common experience of participation in the same research project.^11–13^

### Recruitment and study population

SNAC-K was initiated in 2001 using an age-stratified sampling design. The population of Kungsholmen, a central district of Stockholm, was stratified by age, and random samples were drawn from each age cohort: 60, 66, 72, 78, 81, 84, 87, 90, 93, 96 and 99 years and older. Between 2001 and 2004, 3363 individuals participated in the baseline examination, representing 73.3% of those eligible.^2^ Participants are reassessed at 6-year intervals in the younger age groups (60–72 years) and at 3-year intervals among those aged 78 and older. New cohorts of individuals aged 60 and/or 81 years are regularly added since wave 3. Each assessment includes clinical examinations, cognitive testing and interviews carried out by physicians, nurses and psychologists.

Recruitment for the present study took place during the SNAC-K proband day held at Karolinska Institutet, Sweden, in October 2023. This event aimed to communicate research findings to SNAC-K participants. Those interested in taking part in the qualitative study were invited to register their name and telephone number at a desk outside the lecture hall. Sixty participants expressed interest and provided information on their age, gender, highest educational level (elementary, high school or university) and the year they enrolled in SNAC-K. This information was used to guide the formation of focus groups.

In the weeks following recruitment, one researcher (NF) contacted participants by telephone to explain the study, respond to questions and schedule a suitable date and time for participation at the Aging Research Center, Karolinska Institutet. Non-responders were reminded through follow-up calls, and participants assigned to a group received a text message reminder the week before their scheduled session.

Participants were purposively sampled to ensure variation in age, sex and educational level. Of the 60 individuals who initially expressed interest, 34 ultimately participated in the focus groups. Among those who did not participate, two could not be reached despite follow-up attempts, six were unavailable on the scheduled dates, three did not attend their scheduled focus group and two expressed a preference for online participation only. The remaining 13 individuals were not invited, as sufficient variation in age, sex and socioeconomic position had been achieved, and the data collected were considered adequate to address the study aim.

Using the data collected at registration, seven focus groups were formed (n=34). Participant characteristics are presented in table 1. In the first round, groups were organised by age (one group of participants>75 years and three groups aged 60–75 years), while ensuring representation of both men and women (with at least one man per group), and variation in educational background. To broaden the diversity of perspectives, a second recruitment round targeted one additional group of participants aged over 75 years, one group with a lower educational level, and one group composed only of men.

**Table 1:** List of focus group participants and their demographic information.

Focus group	Age range (years)	Female	Male	University	High school	Elementary school	Unknown educational level
1 (n=6)	63–73	5	1	5	1	0	0
2 (n=6)	63–73	5	1	4	2	0	0
3 (n=5)	81–88	3	2	4	1	0	0
4 (n=5)	71–73	4	1	5	0	0	0
5 (n=4)	80–86	0	4	3	0	0	1
6 (n=3)	81–88	3	0	2	0	0	1
7 (n=5)	72–82	5	0	0	4	1	0

### Interview guide

A semi-structured interview guide was developed to address the study aim (online supplemental file 2). The interview guide included topics related to a newly published study^14^ and participants’ experiences of being part of SNAC-K. It consisted of three main questions and five follow-up questions, supplemented with prompts to encourage participants to elaborate on their thoughts and experiences. The guide was piloted with three older adults in one group session and one individual interview, conducted by two of the researchers (NF and JA). Minor adjustments were made following the pilot to clarify the wording and order of the questions.

### Data generation

The focus groups were conducted face-to-face between December 2023 and January 2024. Three female researchers with backgrounds in public health (JA, AL) and physiotherapy (NF), all experienced in interviewing older adults, facilitated the sessions in pairs. One researcher (NF) moderated all focus groups, while the others alternated between the roles of co-moderator and observer. None of the researchers had any prior relationship with the participants.

The interviews took place in a quiet room at the Aging Research Center, located near Kungsholmen, to provide a comfortable and accessible setting. On arrival, participants received written information in Swedish, as all were fluent in the language, and a consent form. The purpose of the study, its procedures, and the voluntary nature of participation were explained, and participants had the opportunity to ask questions before providing written informed consent. Focus groups lasted approximately 1.5 hours each, were conducted in Swedish, audio recorded, and transcribed verbatim by a professional third-party service.

### Data analysis

After each focus group discussion, the moderator and the observer discussed their initial impressions and reflections. The transcribed data were analysed using inductive qualitative content analysis^9 10^ to provide a broad description of participants’ experiences of participating in SNAC-K. The analytical process began with the first author reading all transcripts repeatedly to become familiar with the material. Meaning-bearing units were then identified, condensed and labelled with preliminary codes. Codes with similar content were grouped into categories that reflected patterns and variations in the data. The analysis was supported by NVivo (QSR International) to facilitate data organisation and coding.

To ensure trustworthiness, several researchers were involved in the analysis. The first author conducted the initial coding, and AL and JA independently reviewed and compared the analyses to validate and refine the emerging categories. All authors participated in iterative discussions to review the content and labelling of categories, comparing them with the original data until consensus was achieved. The researchers brought perspectives from physiotherapy, public health, epidemiology and ageing research. None of the researchers belonged to the population under study, providing an outsider perspective that may have helped them question assumptions and avoid taking participants’ experiences for granted. Throughout the analysis, the team also reflected on how their disciplinary backgrounds and prior experiences might shape data interpretation, and alternative perspectives were actively discussed.

### Patient and public involvement

Recruitment for this study took place during a dissemination event for the SNAC-K cohort (proband day). However, SNAC-K participants were not involved in the design, conduct, analysis or reporting of this specific study.

## Results

A total of 34 participants took part in seven focus group interviews. Participants were aged 63–88 years, 25 (74%) were women and 9 (26%) were men. Most participants had a university education (n=23, 68%), while 11 (32%) had a lower or unknown educational level (table 1).

As shown in figure 1, the analyses generated two main categories *“Blurred boundaries between research and health care”* and *“Participation in research as meaningful yet demanding”*. The categories were built on several related subcategories.

**Figure 1:**
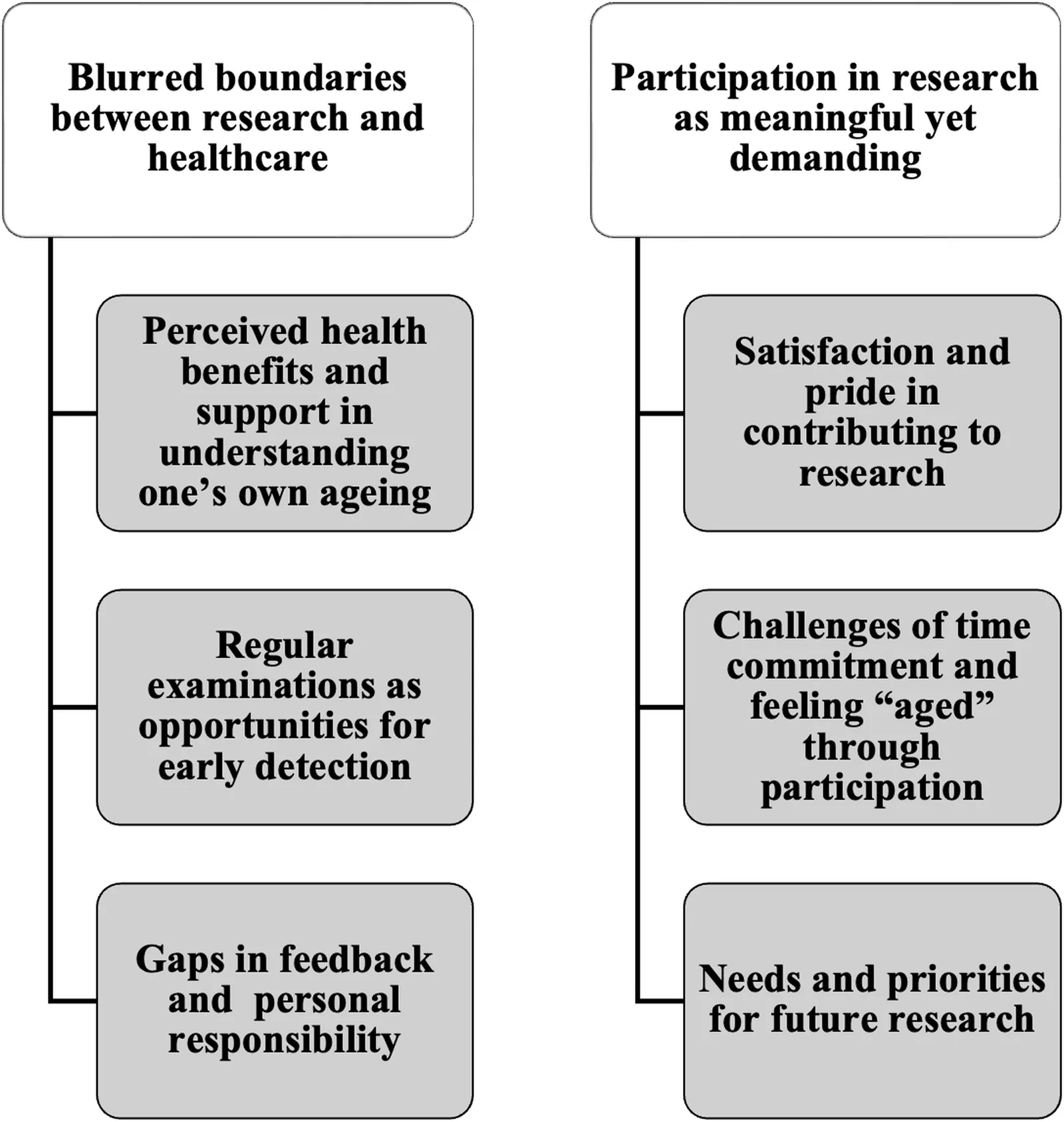
Overview of categories and subcategories derived from the analysis.

### Blurred boundaries between research and health care

Participants appreciated that their involvement in the population-based SNAC-K study provided opportunities for more comprehensive and thorough examinations than those routinely available in primary care. They described this experience as both reassuring and valuable. At the same time, participation also entailed challenges, as the responsibility for acting on unexpected findings and ensuring follow-up within ordinary health services largely rested on the participants themselves.

#### Perceived health benefits and support in understanding one’s own ageing

Participants underlined that they experienced health benefits from participating in SNAC-K. Several expressed gratitude, describing the experience as more personal and rewarding than regular healthcare visits: *I think it’s a stroke of luck to have been selected* (FG5, 80–86 years) and *I think I get a lot personally from being examined in this way at regular intervals* (FG5, 80–86 years).

The opportunity to meet different healthcare professionals within the same context and over time was also seen as an advantage compared with ordinary health care: *I met both a doctor and a nurse. They were just lovely—those are the kind of people I’d like to have in regular care* (FG2, 63–73 years). Participants further highlighted that taking part in SNAC-K offered guidance regarding their own health status. Many valued the repeated assessments and follow-ups over time: *You get a good picture of your own development through all these tests. I can see that my memory has gotten worse compared to the first assessments…* (FG2, 63–73 years).

#### Regular examinations as opportunities for early detection

The interviews also revealed that the comprehensive examinations in SNAC-K had, in several cases, led to the detection of previously unrecognised conditions or deficiencies. Participants described this as important, as it enabled early treatment and potentially prevented future health consequences: *I had really low B12 levels…Looking back, I know that if this hadn’t come to light and I had continued with that deficiency, it could have caused permanent damage* (FG2, 63–73 years). Others described how participation motivated lifestyle changes: *Early in the examinations, I was told that I had too much sugar in my blood. And that was great, because now I live differently* (FG7, 72–82 years).

At the same time, participants expressed a sense of ambivalence, as information received during examinations could also be perceived as misleading or even distressing: *You have a lot that is unknown…but you should probably contact someone…So I went to my GP. He said, ‘no problem, that’s exactly what it looks like for someone your age’. But that was really the only negative part* (FG7, 72–82 years). Despite such experiences, many participants emphasised that the possibility of referral for further assessment contributed to a sense of security: *And if they found something, they would refer you. And I think that feels reassuring and good to have in the background* (FG1, 63–73 years).

#### Gaps in feedback and personal responsibility

While participants appreciated the health-related benefits of participating in SNAC-K, they also described challenges related to follow-up and personal responsibility. One recurring experience was a sense of insufficient follow-up within primary care regarding issues detected during SNAC-K visits.

Another criticism concerned the limited feedback provided after SNAC-K assessments. Many participants expressed a desire to receive more detailed information, particularly regarding cognitive testing: *It’s only for the memory assessments that they don’t provide the results* (FG7, 72–82 years). Others voiced frustration over inconsistencies in communication, noting that some participants appeared to receive more information than others: *What I find strange is that you [another participant] have test results to everything. I haven’t received any test results at all* (FG3, 81–88 years).

Some participants explained that they only received general information and lacked more detailed or continuous feedback to help them interpret their results. Several wished that feedback from the cognitive assessments resembled the more comprehensive responses provided after the physical examinations. Others highlighted that even when feedback was provided, it still required substantial personal interpretation: *You receive a list of your different values [test results], but apart from that I don’t really feel like you get feedback on your health status directly* (FG7, 72–82 years).

Despite these shortcomings, several participants emphasised that participation in SNAC-K did not relieve them of personal responsibility for their health. On the contrary, they stressed the importance of independently seeking care when needed: *You can get sick from one day to the next. And you should seek care anyway, even if you are part of SNAC-K* (FG2, 63–73 years).

### Participation in research as meaningful yet demanding

Participants described both rewarding and challenging aspects of their participation in SNAC-K. While many expressed gratitude and joy at being part of the study, others noted the demanding nature of the assessments and the time commitment involved. Overall, participation was perceived as meaningful, offering a sense of pride and contribution to science and society, while also prompting reflections on ageing, motivation and future research priorities.

#### Satisfaction and pride in contributing to research

Participants expressed a strong interest in the research findings generated by SNAC-K and described the feedback received during proband days held every three years as both motivating and rewarding. The lectures and dissemination activities were perceived as highly stimulating: *It’s fun to be part of it… A whole day we were invited. It was very interesting and rewarding* (FG1, 63–73 years). These gatherings were particularly appreciated as they made complex research findings accessible: *But it’s also fantastic when you go to these gatherings in the auditorium, where knowledgeable people summarise [the findings] in a simple, clear, and good way. Then you feel that this is something I’ve contributed to, that they’ve been able to produce these numbers* (FG5, 80–86 years). Another participant emphasised the value of following the research over time: *I have been involved since the beginning, and I find these recurring reports with doctoral dissertations and such very interesting*… (FG3, 81–88 years).

Beyond interest in the findings themselves, participants described their involvement in SNAC-K as profoundly meaningful because it represented a contribution to society and scientific knowledge: *In general, research is very important. I am very research-oriented and I think it’s important to conduct studies on various things. I think it’s very positive* (FG1, 63–73 years). For some, the value lay in the potential of research to improve health care and treatment: *The results that emerge can lead to better treatments, diagnoses, because it’s not only about ageing…* (FG4, 71–73 years).

Participants further highlighted the importance of making research more accessible and less intimidating: *Just that there’s nothing terrible about talking to a researcher…To de-dramatize research a bit more* (FG1, 63–73 years).

#### Challenges of time commitment and feeling ‘aged’ through participation

Despite many positive experiences, some participants described several challenges related to the assessments. Cognitive and memory tests were frequently perceived as demanding. One participant described the examinations as lengthy and requiring sustained concentration: *They are very demanding. And you do it all at once… And I think that can be very demanding, so that I can’t stay focused* (FG2, 63–73 years). Another participant explained difficulties with memory tasks: *I found it difficult when you had to remember things* (FG6, 81–88 years). For some, these tests also triggered concerns about their own cognitive abilities: *And it was much worse the last time…* (FG7, 72–82 years). Other components of the cognitive assessments, including tests of olfactory function, were also experienced as challenging: *But what was surprisingly difficult, something you wouldn’t expect…was the odors. I mean, is it cinnamon or cardamom?* (FG6, 81–88 years). Some examinations could also be demanding, particularly magnetic resonance imaging (MRI): *Mm, I remember that. Lying completely still. That was tough as hell, I think* (FG7, 72–82 years).

Participants further described the extensive time commitment and questionnaires as burdensome: *I was there for almost four hours yesterday. And then I still needed to come back to do additional parts* (FG2, 63–73 years). Another participant explained that the process was particularly demanding due to a complex medical history: *And it did take quite a long time, especially since I had this long medical history* (FG2, 63–73 years). Questionnaires were at times perceived as repetitive or tedious, although participants accepted this as part of contributing to research: *And then you fill in that whole questionnaire that is so tedious. But you go along with it just because it’s for research* (FG5, 80–86 years).

Participants also prompted reflections related to ageing and age identity. Some participants described initial resistance to participation because the invitation itself made them feel labelled as old: *When I received the letter when I turned 60, I got really annoyed* (FG1, 63–73 years). Another participant similarly reflected: *No, 60. I thought, I’m not that old* (FG1, 63–73 years).

#### Needs and priorities for future research

Participants emphasised the importance of disseminating research results, as well as the need to address future societal challenges related to ageing. Several highlighted the societal and political relevance of SNAC-K: *Politicians should actually learn more from SNAC-K* (FG2, 63–73 years). Another participant reflected on the study’s broader contribution to ageing research and elder care: *If we hadn’t had SNAC-K, how much worse would elder care and ageing research have been in Sweden, and how much better have we become compared to other countries?* (FG3, 81–88 years).

Participants identified several areas they considered important for future research. Social relationships, loneliness, and housing were frequently emphasised: *And there will be more and more who become lonely. And this is a huge issue* (FG7, 72–82 years). Others stressed that the project should address sensitive or overlooked health topics, including suicidality: *I mean, there may be many signs that you are suicidal even if you don’t express it, I think… I believe SNAC-K has a responsibility there* (FG1, 63–73 years). Sexuality was also mentioned as an underexplored aspect of ageing: *Yes, the only thing missing [in the surveys] is the sexual part* (FG1, 63–73 years).

Technology and digital competence were additionally considered increasingly important areas of research in later life: *The technical knowledge of people. Internet skills, Bank-ID. Being able to conduct your errands online…How do we older adults manage?* (FG3, 81–88 years).

Participants also raised existential dimensions related to ageing, life and death: *Yes, life and death and existence. Losses and additions. The differences between generations, and so on* (FG3, 81–88 years). Another participant added: *The final stage of life and death. And death, then it’s the issue of euthanasia. That question* (FG5, 80–86 years).

Finally, participants further noted the importance of addressing disease screening (eg, for osteoporosis), overmedication, elder care, nutrition, economic insecurity, pensions and mental health in future research: *That you get the nutrients you need and not a whole lot of unhealthy things. And with food, it also includes alcohol habits, of course* (FG5, 80–86 years) and *Yes, curiosity and quality of life and mental health* (FG6, 81–88 years).

## Discussion

This study explored older adults’ experiences of participating in a longitudinal population-based study on ageing and care, SNAC-K. The findings show that participation in SNAC-K was perceived as both valuable and demanding. Two main categories emerged: *blurred boundaries between research and health care* and *participation as meaningful yet demanding*.

Participants described several health-related benefits of participation, including access to comprehensive examinations, repeated follow-ups and, in some cases, early detection of previously unrecognised conditions. These experiences were often framed as reassuring and worthwhile, particularly compared with regular healthcare contacts. Similar motivations and perceived benefits have been reported in previous studies, where older adults described participation in longitudinal research as worth the effort and meaningful for personal reasons.^3 4^ At the same time, participants’ accounts can be understood in relation to broader transformations in Swedish health care, where responsibility for monitoring and maintaining health has increasingly shifted towards the individual. While individual actions in promoting health have long been acknowledged, the late 20th century saw a normative shift towards expecting individuals to assume greater responsibility for their own health.^15 16^ In this context, participants’ appreciation of the regular assessments within SNAC-K may reflect perceived gaps in routine care, particularly regarding continuity, monitoring and preventive follow-up. This interpretation aligns with research highlighting unmet care and support needs among older adults, especially in relation to guidance, coordination and ongoing support.^17^ In parallel, these findings may also be understood in relation to broader societal processes of medicalisation, whereby health care is increasingly expected to monitor, interpret and provide reassurance regarding experiences associated with ageing and everyday life.^18^ This raises important concerns, as observational studies are not intended to compensate for deficiencies in routine healthcare provision or to replace adequate support and follow-up within public health services.

Participants also expressed a desire for more consistent and detailed feedback, particularly regarding cognitive assessments, and described situations where responsibility for follow-up rested on themselves. These findings point to a potential tension inherent in longitudinal observational research.^3^ Although studies such as SNAC-K generate health-related information and have ethical and legal obligations to act on unexpected findings suggestive of potentially serious health conditions, their primary scientific purpose is to observe ageing trajectories and health changes over time rather than to intervene or provide clinical management. Extensive individualised feedback may therefore risk altering the observational nature of the study and influence participants’ behaviours, health-seeking patterns or experiences of ageing. Still, participants’ experiences suggest that health-related findings may generate expectations resembling those associated with healthcare encounters, raising ethical concerns regarding therapeutic misconception, whereby research activities are interpreted as clinical care.^19^ Maintaining clear boundaries between research and health care may therefore represent an ongoing challenge for researchers and data collection staff, particularly in long-term studies involving repeated physiological and cognitive assessments. Together, these findings highlight the importance of clear and transparent communication regarding the scope, limits and purpose of feedback within observational research.

Participants described their involvement as meaningful, emphasising a sense of contributing to research and society. This can be understood within the broader framework of patient and public involvement, where participants are increasingly viewed as active contributors rather than passive subjects.^8^ In the present study, participants expressed forms of engagement that extended beyond passive participation, particularly through their interest in research findings, reflections on research priorities and sense of contributing to scientific knowledge. At the same time, achieving such engagement in practice is not without challenges. Previous research has highlighted that clarifying roles and expectations is central to achieving meaningful engagement, as ambiguity may lead to mismatched expectations, challenges in collaboration and uncertainty regarding how such involvement should be operationalised.^20–22^ The proband days in SNAC-K appeared to function as a concrete mechanism for fostering participant engagement and reciprocal communication, strengthening participants’ sense of contribution and connection to the research. Nevertheless, participant involvement within SNAC-K could potentially be further developed beyond communication and consultation, for example, through more active roles in shaping research priorities and dissemination activities.

Despite these positive experiences, participants also described several challenges related to participation. The assessments were sometimes experienced as time-consuming and demanding, particularly the cognitive tests and extensive questionnaires. In addition, some participants reported that cognitive testing could evoke concerns about their own abilities and ageing. These findings align with previous research indicating that participation in longitudinal studies can involve both positive and negative experiences, including fatigue, discomfort and emotional reactions.^3 4^ Prior studies have similarly shown that repeated questioning, limited feedback and demanding assessments may negatively shape participants’ experiences,^23^ especially among older individuals and those with lower cognitive or educational resources.^3^ It is important to acknowledge that SNAC-K, like other longitudinal studies involving extensive assessments, undergoes regular ethical review to ensure that scientific benefits outweigh potential participant burden and discomfort. Research staff are trained to minimise distress and avoid reinforcing ageist attitudes, and participants are free to withdraw at any time. Nevertheless, these findings remain important for the research community, as they highlight the personal effort involved in long-term research participation and the need to continuously reflect on how to minimise burden while maintaining high-quality research standards.

Finally, participants highlighted several areas they considered important for future research, including loneliness, mental health, housing, digital competence and existential aspects of ageing. These priorities reflect a broader understanding of healthy ageing, in which well-being in later life is shaped not only by physical and mental capacities but also by social relationships, housing conditions and the environments in which older adults live.^1^ By extending beyond biomedical perspectives, these findings support a broader understanding of health as the ability to live a meaningful and valued life in old age. This aligns with the capability-based perspective on healthy ageing, which emphasises individuals’ real opportunities to achieve well-being within their specific contexts.^24^

### Study limitations and strengths

This study has several limitations that should be considered when interpreting the findings. First, recruitment took place during a proband day, meaning that only individuals who were able and willing to attend this event were eligible for inclusion. This may have resulted in a sample skewed towards more motivated and engaged participants, while more frail individuals, those with cognitive impairment or those with mobility limitations may have been underrepresented. In addition, the study was conducted in an urban Swedish context, and participants were largely drawn from a relatively socioeconomically advantaged area, which may limit the transferability of the findings to other populations or settings. Second, although focus groups can facilitate reflection and interaction, they may also limit the expression of more critical or sensitive experiences. Moreover, participants who are more comfortable speaking in groups or who are more socially connected may have been more likely to participate.^25^ The findings were not returned to participants for feedback or verification but evidence regarding its contribution to validity in qualitative research remains mixed.^26^ Lastly, the researchers’ backgrounds in ageing and health care may also have influenced the analysis. To address this, multiple researchers were involved in the analytic process, and interpretations were continuously discussed and compared with the original data.

The study also has several strengths. It provides in-depth insights into participants’ lived experiences of long-term involvement in a population-based ageing study, a perspective that has been rarely explored. Variation in age, gender and educational background across focus groups contributed to capturing a range of experiences and perspectives. Furthermore, the involvement of several researchers and the iterative analytic process strengthened the credibility of the findings.^27^

## Conclusion

Taken together, the findings suggest that participation in longitudinal ageing research is experienced as both valuable and complex. Participants appreciated the opportunity to contribute to science and gain insights into their own health and ageing. At the same time, the findings highlight the importance of clear communication regarding study aims, scope and feedback procedures, managing expectations, minimising burden and maintaining a clear distinction between research and health care. The study also underscores the value of actively incorporating older adults’ perspectives into the design and conduct of longitudinal ageing research to strengthen both its ethical conduct and societal relevance.

## Supplementary material

10.1136/bmjopen-2026-123537 online supplemental file 1 & 2

## Data Availability

No data are available.

## References

[1] Rudnicka E, Napierała P, Podfigurna A (2020). The World Health Organization (WHO) approach to healthy ageing. Maturitas.

[2] Lagergren M, Fratiglioni L, Hallberg IR (2004). A longitudinal study integrating population, care and social services data. The Swedish National study on Aging and Care (SNAC). Aging Clin Exp Res.

[3] Dahlin-Ivanoff S, Sterner TR, Blennow K (2019). Was it worth it? Older adults’ experiences of participating in a population-based cohort study - a focus group study. BMC Geriatr.

[4] von Strauss E, Fratiglioni L, Jorm AF (1998). Attitudes and Participation of the Elderly in Population Surveys: Data from a Longitudinal Study on Aging and Dementia in Stockholm. J Clin Epidemiol.

[5] Sacristán JA, Aguarón A, Avendaño-Solá C (2016). Patient involvement in clinical research: why, when, and how. Patient Prefer Adherence.

[6] James H, Buffel T (2023). Co-research with older people: a systematic literature review. Ageing Soc.

[7] Baldwin JN, Napier S, Neville S (2018). Impacts of older people’s patient and public involvement in health and social care research: a systematic review. Age Ageing.

[8] Harrington RL, Hanna ML, Oehrlein EM (2020). Defining Patient Engagement in Research: Results of a Systematic Review and Analysis: Report of the ISPOR Patient-Centered Special Interest Group. Value Health.

[9] Graneheim UH, Lindgren BM, Lundman B (2017). Methodological challenges in qualitative content analysis: A discussion paper. Nurse Educ Today.

[10] Elo S, Kääriäinen M, Kanste O (2014). Qualitative Content Analysis: A Focus on Trustworthiness. Sage Open.

[11] Morgan DL (1993). Successful Focus Groups: Advancing the State of the Art.

[12] Morgan DL (1997). Focus Groups as Qualitative Research.

[13] Kvale S (2014). InterViews: Learning the Craft of Qualitative Research Interviewing.

[14] Frisendahl N, Liljas A, Kirvalidze M (2026). Perceptions on healthy aging: insights from focus group interviews of older adults in Sweden. Arch Public Health.

[15] Lupton D (1995). The Imperative of Health: Public Health and the Regulated Body.

[16] Minkler M (1999). Personal responsibility for health? A review of the arguments and the evidence at century’s end. Health Educ Behav.

[17] Abdi S, Spann A, Borilovic J (2019). Understanding the care and support needs of older people: a scoping review and categorisation using the WHO international classification of functioning, disability and health framework (ICF). BMC Geriatr.

[18] Jønsson ABR (2024). Medicalization of Old Age: Experiencing Healthism and Overdiagnosis in a Nordic Welfare State. Med Anthropol.

[19] Henderson GE, Churchill LR, Davis AM (2007). Clinical trials and medical care: defining the therapeutic misconception. PLoS Med.

[20] Smits D-W, van Meeteren K, Klem M (2020). Designing a tool to support patient and public involvement in research projects: the Involvement Matrix. Res Involv Engagem.

[21] Frögren J, Schmidt SM, Kylén M (2022). Awareness of and attitudes towards public involvement in research on ageing and health among older people in Sweden. PLoS ONE.

[22] Kylén M, Slaug B, Jonsson O (2022). User involvement in ageing and health research: a survey of researchers’ and older adults’ perspectives. Health Res Policy Syst.

[23] Naidoo N, Nguyen VT, Ravaud P (2020). The research burden of randomized controlled trial participation: a systematic thematic synthesis of qualitative evidence. BMC Med.

[24] Venkatapuram S, Amuthavalli Thiyagarajan J (2023). The Capability Approach and the WHO healthy ageing framework (for the UN Decade of Healthy Ageing). Age Ageing.

[25] Moser A, Korstjens I (2018). Series: Practical guidance to qualitative research. Part 3: Sampling, data collection and analysis. Eur J Gen Pract.

[26] Thomas DR (2017). Feedback from research participants: are member checks useful in qualitative research?. Qual Res Psychol.

[27] Shenton AK (2004). Strategies for ensuring trustworthiness in qualitative research projects. EFI.

