# Supplementary material for: Older adults’ experiences of participating in longitudinal ageing research: a qualitative study from the Swedish National study on Aging and Care in Kungsholmen
Source: BMJ Open. 2026 Nov 10;16(11):e123537. doi: 10.1136/bmjopen-2026-123537 (PMC13756336; doi:10.1136/bmjopen-2026-123537)
Supplement: Supplementary file 1 [file bmjopen-2026-123537.reviewer_comments.pdf]

PEER REVIEW HISTORY

BMJ Open publishes all reviews undertaken for accepted manuscripts. Reviewers are asked to complete a checklist review form and are provided with free text boxes to elaborate on their assessment. These free text comments are reproduced below.

ARTICLE DETAILS

Title (Provisional)

Older adults' experiences of participating in longitudinal ageing research: A qualitative study from the Swedish National study on Aging and Care in Kungsholmen.

Authors

Frisendahl, Nathalie; Liljas, Ann; Kirvalidze, Mariam; Agerholm, J; Calderón-Larrañaga, Amaia; Welmer, Anna-Karin

---

VERSION 1 - REVIEW

**Reviewer**	**1**
**Name**	**Popa-Wagner, Aurel**
**Affiliation**	**University of Duisburg-Essen**
**Date**	**28-Jul-2026**
**COI**	**None**

---

his qualitative study addresses an important but often overlooked aspect of longitudinal ageing research by examining how older adults perceive their long-term participation in the Swedish National Study on Aging and Care in Kungsholmen (SNAC-K). While population-based ageing cohorts have substantially advanced our understanding of the biological and social determinants of healthy ageing, considerably less attention has been paid to the experiences of the participants who make these studies possible. The findings show that participation in SNAC-K was perceived as both valuable and demanding. Hopefully, the study will offer much more data beyondwhat it does in the current study.

**Reviewer**	**2**
**Name**	**Pankhurst, Morgan**
**Affiliation**	**Flinders University**
**Date**	**04-Sep-2026**
**COI**	**None**

Thank you for the opportunity to review this primary research article. I felt the results were particularly strong and an excellent example of ICA. I have noted below sections that felt a bit unclear.

Page 4, Line 37: The study design is stated as qualitative inductive, this doesn't give the reader of the theoretical approach. Inductive usually refers to data analysis rather than study design. Suggest some additional clarity around the methodological approach. Additionally focus groups are principally a qualitative data-collection method/technique, not a complete research methodology or design.

Page 5, Line 22: Authors refer to table one in the same sentence that they indicate 60 participants registered interest, however Table 1 does not contain the data for 60 participants. There is no description or indication as to why the discrepancy.

Page 5, Line 38: Authors claim that groups were gender balanced (with at least one male per group). I think claiming that including one person of the opposite gender resulted in balance is a bit of an overstatement.

Page 7, Line 5: Authors state 'they' were not involved, it is unclear who they are.

Page 7, Line 28: The results do not start with a description of the participants, although Table 1 provides the breakdown of each focus group, it doesn't clearly summarise the sample.

Page 8, Line 25: Participant quotes are tagged with the focus group number, it might also be helpful to indicate the age and gender of the participant providing the quote. The age range in focus groups is quite large and there is a big difference between how we experience the word at 63 vs 73 or 72 vs 82. This is also important to add context to quotes such as ‘no problem, that’s exactly what it looks like for someone your age’. What age is the GP referring to?

Page 11, Line 27: The quote about smells lacks context, why would the participant be sniffing cinnamon?

The SRQR states that researcher reflexivity and positionality is mentioned on pages 6 and 16. Whilst there is some mention of cross checking and trustworthiness, researcher positionality is not clearly described.

The abstract states: However, many also expressed expectations

regarding feedback, reassurance, and clinical follow-up that exceeded the remit of

observational research, reflecting perceived shortcomings in routine healthcare provision. This doesn't quite align with the discussion which states "participants’ appreciation of the regular assessments within SNAC-K may reflect perceived gaps in routine care". The wording in the abstract links two constructs in a way that is unclear.

VERSION 1 - AUTHOR RESPONSE

Reviewer 1

Dr. Aurel Popa-Wagner, University of Duisburg-Essen
Comments to the Author:
This qualitative study addresses an important but often overlooked aspect of longitudinal ageing research by examining how older adults perceive their long-term participation in the Swedish National study on Aging and Care in Kungsholmen (SNAC-K). While population-based ageing cohorts have substantially advanced our understanding of the biological and social determinants of healthy ageing, considerably less attention has been paid to the experiences of the participants who make these studies possible. The findings show that participation in SNAC-K was perceived as both valuable and demanding. Hopefully, the study will offer much more data beyond what it does in the current study.

Response: We thank the reviewer for the positive and encouraging comments. We appreciate the recognition of the importance of exploring older adults' experiences of participation in longitudinal ageing research and the value of this work for understanding participant perspectives in studies such as SNAC-K.

Reviewer 2

Dr. Morgan Pankhurst, Flinders University
Comments to the Author:
Thank you for the opportunity to review this primary research article. I felt the results were particularly strong and an excellent example of ICA. I have noted below sections that felt a bit unclear.

Response: We thank the reviewer for the thoughtful and constructive feedback and for the positive assessment of our study. We appreciate the reviewer's comments and suggestions and have carefully revised the manuscript to address the points raised below.

Page 4, Line 37: The study design is stated as qualitative inductive, this doesn't give the reader of the theoretical approach. Inductive usually refers to data analysis rather than study design. Suggest some additional clarity around the methodological approach. Additionally focus groups are principally a qualitative data-collection method/technique, not a complete research methodology or design.

Response: We thank the reviewer for this helpful comment. We agree that the previous wording did not clearly distinguish between study design, data collection method, and analytical approach. The manuscript has been revised to clarify that a qualitative study design was employed and that data were collected through focus group interviews. The reference to an inductive approach has been removed from the Study Design section, as this pertains to the analytical process and is already described in the Data Analysis section. (page 4, line 100-101,103)

Page 5, Line 22: Authors refer to table one in the same sentence that they indicate 60 participants registered interest, however Table 1 does not contain the data for 60 participants. There is no description or indication as to why the discrepancy.

Response: We thank the reviewer for this comment. We agree that the previous wording was unclear. Table 1 describes the characteristics of the 34 participants included in the focus groups, not the 60 individuals who initially expressed interest. We have therefore removed the table reference from the recruitment description and relocated it to the section presenting the final study sample. (page 5, lines 134-135) In addition, we have clarified the discrepancy between the number of individuals who initially expressed interest and those ultimately included in the study. We now provide a more detailed account of the recruitment and sampling process, specifying the number of participants who could not be reached, were unavailable, did not attend their scheduled focus group, or expressed a preference for online participation only. We also clarify that some individuals were not invited because sufficientvariation in age, sex and socioeconomic position had already been achieved, and the data collected was considered adequate to address the study aim. (page 5, lines 127-133)

Page 5, Line 38: Authors claim that groups were gender balanced (with at least one male per group). I think claiming that including one person of the opposite gender resulted in balance is a bit of an overstatement.

Response: We thank the reviewer for this observation. We agree that the term "gender balance" may overstate the composition of the focus groups. To improve accuracy, we have replaced "balance of gender" with "representation of both men and women" to better reflect our sampling strategy and group composition. (page 5, line 137)

Page 7, Line 5: Authors state 'they' were not involved, it is unclear who they are.

Response: We thank the reviewer for identifying this ambiguity. We agree that the pronoun "they" was unclear. To improve clarity, we have replaced it with "SNAC-K participants" to explicitly identify the group being referred to. (page 7, 186)

Page 7, Line 28: The results do not start with a description of the participants, although Table 1 provides the breakdown of each focus group, it doesn't clearly summarise the sample.

Response: We thank the reviewer for this helpful comment. We agree that the participant sample was not sufficiently described at the beginning of the Results section. We have therefore added a brief summary of the study sample, including the number of focus groups, participant age range, gender distribution, and educational background, while retaining the detailed information in Table 1. (page 7, 197-199)

Page 8, Line 25: Participant quotes are tagged with the focus group number, it might also be helpful to indicate the age and gender of the participant providing the quote. The age range in focus groups is quite large and there is a big difference between how we experience the word at 63 vs 73 or 72 vs 82. This is

also important to add context to quotes such as ‘no problem, that’s exactly what it looks like for someone your age’. What age is the GP referring to?

Response: We thank the reviewer for this thoughtful suggestion. We agree that participant characteristics can provide useful context for the interpretation of quotations. However, in focus group research it is not always possible to reliably attribute quotations to specific individuals while maintaining confidentiality. In other words, providing both age and gender for individual quotations could increase the risk of participant identification, particularly given the relatively small sample and focus group composition. We therefore chose not to report individual-level characteristics alongside quotations. To provide additional context while preserving anonymity, we have revised the manuscript to include the age range of the focus group together with each quotation. (page 8-13)

Page 11, Line 27: The quote about smells lacks context, why would the participant be sniffing cinnamon?

Response: We thank the reviewer for this comment. We agree that the text could be interpreted ambiguously. To improve clarity, we have revised the wording to specify that the quotation refers to another component of the cognitive assessments, including tests of olfactory function, thereby making the context of the participant's statement clearer. (page 11, line 306)

The SRQR states that researcher reflexivity and positionality is mentioned on pages 6 and 16. Whilst there is some mention of cross checking and trustworthiness, researcher positionality is not clearly described.

Response: We thank the reviewer for this important observation. While information regarding the researchers' backgrounds and procedures to enhance trustworthiness was already included in the manuscript, we agree that researcher positionality was not addressed explicitly. We have therefore added a brief statement outlining the researchers' disciplinary backgrounds, their outsider perspective, and the reflexive discussions undertaken throughout the analytical process to critically consider how their prior experiences and professional perspectives might have influenced data interpretation. (page 7, line 176-182)

The abstract states: However, many also expressed expectations regarding feedback, reassurance, and clinical follow-up that exceeded the remit of observational research, reflecting perceived shortcomings in routine healthcare provision. This doesn't quite align with the discussion which states "participants’ appreciation of the regular assessments within SNAC-K may reflect perceived gaps in routine care". The wording in the abstract links two constructs in a way that is unclear.

Response: We thank the reviewer for this helpful observation. To improve consistency with the Discussion section, we have revised the abstract to use more cautious wording and align it with our interpretation that participants’ appreciation of the regular assessments within SNAC-K may reflect perceived gaps in routine healthcare provision. (page 2, line 43-44)

VERSION 2 - REVIEW

**Reviewer**	**2**
**Name**	**Pankhurst, Morgan**
**Affiliation**	**Flinders University**
**Date**	**22-Oct-2026**
**COI**	

Thank you for the care and attention you have given to the recommendations made in the previous review. I am happy to accept this on the provision that citations are carefully checked against claims. For example, "older adults' own perspectives on research participation remain relatively understudied [3]" the citation [Clarke P, Nieuwenhuijsen ER. Environments for healthy ageing: a critical review. Maturitas. 2009;64(1):14–19.] is a review of environmental influences on healthy ageing, focussing on built environments, neighbourhoods, mobility and aging rather than research participation or experiences.

VERSION 2 - AUTHOR RESPONSE

Reviewer 2

Dr. Morgan Pankhurst, Flinders University
Comments to the Author:
Thank you for the care and attention you have given to the recommendations made in the previous review. I am happy to accept this on the provision that citations are carefully checked against claims. For example, "older adults' own perspectives on research participation remain relatively understudied [3]" the citation [Clarke P, Nieuwenhuijsen ER. Environments for healthy ageing: a critical review. Maturitas. 2009;64(1):14–19.] is a review of environmental influences on healthy ageing, focussing on built environments, neighbourhoods, mobility and aging rather than research participation or experiences.

Response:
We thank the reviewer for identifying this issue. We agree that the cited reference was not appropriate to support the statement regarding older adults' perspectives on research participation. The reference by Clarke and Nieuwenhuijsen has therefore been removed from the manuscript. The statement is now supported by studies specifically examining older adults' experiences of participation in longitudinal ageing research (Dahlin-Ivanoff et al., 2019; von Strauss et al., 1998). We have also carefully reviewed all citations throughout the manuscript to ensure that references accurately support the associated claims.
