# Supplementary material for: Older adults’ experiences of participating in longitudinal ageing research: a qualitative study from the Swedish National study on Aging and Care in Kungsholmen
Source: BMJ Open. 2026 Nov 10;16(11):e123537. doi: 10.1136/bmjopen-2026-123537 (PMC13756336; doi:10.1136/bmjopen-2026-123537)
Supplement: Supplementary file 2 [file bmjopen-16-11-s001.docx]

**Supplemantery file 1.** Standards for Reporting Qualitative Research.

|  | **Standards for Reporting Qualitative Research (SRQR)*** |  |
| --- | --- | --- |
|  | <http://www.equator-network.org/reporting-guidelines/srqr/> |  |
|  |  | **Page/line no(s).** |
| **Title and abstract** | |  |
|  | **Title** - Concise description of the nature and topic of the study Identifying the study as qualitative or indicating the approach (e.g., ethnography, grounded theory) or data collection methods (e.g., interview, focus group) is recommended | Page 1 |
|  | **Abstract** - Summary of key elements of the study using the abstract format of the intended publication; typically includes background, purpose, methods, results, and conclusions | Page 2 |
|  |  |  |
| **Introduction** | |  |
|  | **Problem formulation** - Description and significance of the problem/phenomenon studied; review of relevant theory and empirical work; problem statement | Page 3 |
|  | **Purpose or research questio**n - Purpose of the study and specific objectives or questions | Page 4 |
|  |  |  |
| **Methods** | |  |
|  | **Qualitative approach and research paradigm** - Qualitative approach (e.g., ethnography, grounded theory, case study, phenomenology, narrative research) and guiding theory if appropriate; identifying the research paradigm (e.g., postpositivist, constructivist/ interpretivist) is also recommended; rationale** | Page 4 |
|  | **Researcher characteristics and reflexivity** - Researchers’ characteristics that may influence the research, including personal attributes, qualifications/experience, relationship with participants, assumptions, and/or presuppositions; potential or actual interaction between researchers’ characteristics and the research questions, approach, methods, results, and/or transferability | Page 6-7, 16 |
|  | **Context** - Setting/site and salient contextual factors; rationale** | Page 5 |
|  | **Sampling strategy** - How and why research participants, documents, or events were selected; criteria for deciding when no further sampling was necessary (e.g., sampling saturation); rationale** | Pages 4-5 |
|  | **Ethical issues pertaining to human subjects** - Documentation of approval by an appropriate ethics review board and participant consent, or explanation for lack thereof; other confidentiality and data security issues | Page 7 |
|  | **Data collection methods** - Types of data collected; details of data collection procedures including (as appropriate) start and stop dates of data collection and analysis, iterative process, triangulation of sources/methods, and modification of procedures in response to evolving study findings; rationale** | Pages 4-6 |
|  | **Data collection instruments and technologies** - Description of instruments (e.g., interview guides, questionnaires) and devices (e.g., audio recorders) used for data collection; if/how the instrument(s) changed over the course of the study | Page 5-6 |
|  | **Units of study** - Number and relevant characteristics of participants, documents, or events included in the study; level of participation (could be reported in results) | Page 4-5 |
|  | **Data processing** - Methods for processing data prior to and during analysis, including transcription, data entry, data management and security, verification of data integrity, data coding, and anonymization/de-identification of excerpts | Page 6-7 |
|  | **Data analysis** - Process by which inferences, themes, etc., were identified and developed, including the researchers involved in data analysis; usually references a specific paradigm or approach; rationale** | Page 6-7 |
|  | **Techniques to enhance trustworthiness** - Techniques to enhance trustworthiness and credibility of data analysis (e.g., member checking, audit trail, triangulation); rationale** | Page 6-7, 16 |
|  |  |  |
| **Results/findings** | |  |
|  | **Synthesis and interpretation** - Main findings (e.g., interpretations, inferences, and themes); might include development of a theory or model, or integration with prior research or theory | Pages 7-13 |
|  | **Links to empirical data** - Evidence (e.g., quotes, field notes, text excerpts, photographs) to substantiate analytic findings | Pages 8-13 |
|  |  |  |
| **Discussion** | |  |
|  | **Integration with prior work, implications, transferability, and contribution(s) to the field -** Short summary of main findings; explanation of how findings and conclusions connect to, support, elaborate on, or challenge conclusions of earlier scholarship; discussion of scope of application/generalizability; identification of unique contribution(s) to scholarship in a discipline or field | Pages 13-16 |
|  | **Limitations** - Trustworthiness and limitations of findings | Page 16 |
|  |  |  |
| **Other** | |  |
|  | **Conflicts of interest** - Potential sources of influence or perceived influence on study conduct and conclusions; how these were managed | Page 17 |
|  | **Funding** - Sources of funding and other support; role of funders in data collection, interpretation, and reporting | Page 17-18 |
|  |  |  |
|  | *The authors created the SRQR by searching the literature to identify guidelines, reporting standards, and critical appraisal criteria for qualitative research; reviewing the reference lists of retrieved sources; and contacting experts to gain feedback. The SRQR aims to improve the transparency of all aspects of qualitative research by providing clear standards for reporting qualitative research. |  |
|  |  |  |
|  | **The rationale should briefly discuss the justification for choosing that theory, approach, method, or technique rather than other options available, the assumptions and limitations implicit in those choices, and how those choices influence study conclusions and transferability. As appropriate, the rationale for several items might be discussed together. |  |
|  |  |  |
|  | **Reference:** |  |
|  | O'Brien BC, Harris IB, Beckman TJ, Reed DA, Cook DA. **Standards for reporting qualitative research: a synthesis of recommendations.** *Academic Medicine*, Vol. 89, No. 9 / Sept 2014  DOI: 10.1097/ACM.0000000000000388 |  |

**Supplementary file 2.** Semi-structured interview guide.

**Semi-structured interview guide for focus group interviews**

Thank you very much for coming here today! My name is XXX and I will be leading this interview, and this is XXX, who will be acting as an observer (and thus taking some notes) throughout the interview.

The purpose of our project is to learn about people's experiences of aging, what is important during that process and how to imagine your future when you become old. We also want to address how participants in the SNAC-K study experience being part of the study, and what you think about the information being collected.

We have invited you here today to hear your experiences and thoughts on the matter. I'm going to ask a few questions and then you can add more at the end of the interview if anything comes up.

- Provide the informed consent document.
- Explain the importance and authorization to record the interview, insisting on the confidentiality of the information.
- Inform about the pseudonymized document that will be used for collecting sociodemographic data as well as the participation time in the SNAC-K study.
- Participants’ answers are followed up with questions such as: Do you wish to tell me more about this? What is this due to? What is missing? What is needed?

**PART I: PEOPLE'S EXPERIENCES WITH AGING**

1. What does healthy aging mean to you? (feel free to take a couple of minutes to think about this question)

NOTE: if they do not respond spontaneously, you may personalize the question to their own cases: *How would you like to age? How would you like your old age to look like from now on?*

2. What do you think it takes to be able to age the way you want?

NOTE I: if they do not respond spontaneously, you may relate this question to question 1: *Of the aspects that you have mentioned that influence healthy aging, which would you consider the most important and why?*

NOTE II: by “aspects” we mean help, tools, strategies of any kind, personal, family- or community-related, etc.

3. What possible obstacles prevent you from aging the way you want?

NOTE I: if they do not respond spontaneously, you may relate this question to question 1: *Of the aspects that you have mentioned that influence healthy aging, do you personally think you have such resources? Which ones do you miss most?*

NOTE II: by obstacles we mean difficulties that may be encountered of all kinds, personal, family-related, social, architectural, economic, technology or digitalization-related, the Covid pandemic, etc.

**PART II: SNAC-K PARTICIPANTS’ EXPERIENCE BEING PART OF THE STUDY**

4. How do you see the SNAC-K study of which you are part?

NOTE: if they do not respond spontaneously, you may ask: *Why did you join the study? What are the main reasons why you continue? Do you think other participants’ experiences are similar to yours?*

5. Do you think that the SNAC-K study satisfactorily captures those dimensions or aspects that we discussed earlier about how we understand healthy aging? Why or why not?

NOTE: additional related questions: *Which of those aspects are collected satisfactorily and which ones are missing or could be collected better?*

6. Do you have any other thoughts that you want to highlight?

**END OF INTERVIEW**

- Finish by thanking them again for their participation, asking how they felt and informing them about our intention to contact them again to send them a draft of the provisional results (by email).
- The moderator and the observer will meet once the participants leave to fill in the notebook with their impressions about the focus group (e.g. level of participation, possible preliminary findings, what was expected to be discussed and did not arise and vice versa).

**Semistrukturerad intervjuguide för fokusgruppsintervjuer**

Tack så mycket för att ni kom hit idag! Jag heter XXX och jag kommer att leda den här intervjun, och det här är XXX, som kommer att agera som observatör (och ta anteckningar) under intervjun.

Syftet med det här projektet är att vi vill lära oss om människors upplevelser av åldrandet, vad som är viktigt under den processen och hur man föreställer sig sin framtid när man blir gammal. Vi vill också ta upp hur deltagare i SNAC-K-studien upplever att vara en del av studien, och vad ni tycker om informationen som samlas in.

Vi har bjudit in er hit idag för att höra era erfarenheter och tankar om detta. Jag kommer att ställa några frågor och sedan kan ni lägga till fler i slutet av intervjun om något dyker upp.

- Tillhandahåll dokumentet för informerat samtycke.

- Förklara vikten och godkännandet av att spela in intervjun och förtydliga att informationen är konfidentiell.

- Informera om det pseudonymiserade dokumentet som kommer att användas för insamling av sociodemografisk data samt deltagandetiden i SNAC-K-studien.

– Deltagarnas svar följs upp med frågor som: Vill du berätta mer om detta? Vad beror detta på? Vad saknas? Vad behövs?

**DEL I: MÄNNISKORS ERFARENHETER AV ÅLDRANDE**

1. Vad betyder hälsosamt åldrande för dig? (ta gärna ett par minuter att fundera över denna fråga)

OBS: om de inte svarar spontant kan du anpassa frågan till deras egna fall: Hur skulle du vilja åldras? Hur skulle du vilja att din ålderdom skulle se ut från och med nu?

2. Vad tror du att det krävs för att kunna åldras som du vill?

OBS I: om de inte svarar spontant kan du relatera denna fråga till fråga 1: Av de aspekter som du har nämnt som påverkar hälsosamt åldrande, vilken skulle du anse vara viktigast och varför? OBS II: med "aspekter" menar vi hjälp, verktyg, strategier av alla slag, personliga, familje- eller samhällsrelaterade, etc.

3. Vilka eventuella hinder skulle kunna hindra dig från att åldras som du vill?

OBS I: om de inte svarar spontant kan du relatera denna fråga till fråga 1: Av de aspekter som du har nämnt som påverkar hälsosamt åldrande, tror du att du har sådana resurser? Om inte, vilka saknar du mest?

OBS II: med hinder menar vi svårigheter som kan uppstå av alla slag, personliga, familjerelaterade, sociala, arkitektoniska, ekonomiska, teknologiska eller digitaliseringsrelaterade, Covid-pandemin, etc.

**DEL II: SNAC-K-DELTAGARES ERFARENHET ATT ÄR EN DEL AV STUDIEN**

4. Hur ser du på SNAC-K-studien som du är en del av?

OBS: om de inte svarar spontant kan du fråga: Varför gick du med i studien? Vilka är de främsta anledningarna till att du fortsätter? Tror du att andra deltagares erfarenheter liknar dina?

5. Anser du att SNAC-K-studien fångar de dimensioner eller aspekter som vi diskuterade tidigare om hur vi förstår hälsosamt åldrande? Varför eller varför inte?

OBS: ytterligare relaterade frågor: Vilka av dessa aspekter samlas in på ett tillfredsställande sätt och vilka saknas eller skulle kunna samlas in bättre?

6. Har du några andra tankar som du vill lyfta fram?

**SLUT PÅ INTERVJU**N

- Avsluta med att tacka dem igen för deras deltagande, fråga hur de upplevde intervjun och informera dem om vår avsikt att kontakta dem igen för att skicka dem ett utkast till de preliminära resultaten (via e-post).

- Moderatorn och observatören kommer att träffas när deltagarna lämnar lokalen för att fylla i anteckningsboken med sina intryck av fokusgruppen (t.ex. deltagandegrad, möjliga preliminära resultat, vad som förväntades diskuteras och inte kom upp och vice versa).
